# A Hybrid Intelligent Fault Diagnosis Framework for Rolling Bearings and Gears Based on BAYES-ICEEMDAN-SNR Feature Enhancement and ITOC-LSSVM

**DOI:** 10.3390/s26051543

**Published:** 2026-02-28

**Authors:** Xiaoxu He, Xingwei Ge, Zhe Wu, Qiang Zhang, Yiying Yang, Yachao Cao

**Affiliations:** 1 School of Mechanical Engineering, Hebei University of Science and Technology, Shijiazhuang 050018, China; 18000310722@163.com (X.H.); wuzhe@hebust.edu.cn (Z.W.); yachao.cao@hebust.edu.cn (Y.C.); 2 Key Laboratory of Vehicle Transmission, China North Vehicle Research Institute, Beijing 100072, China; giangzh36@gmail.com; 3 Hebei Technology Innovation Center for Clean and Efficient Thermal Power Cogeneration, Shijiazhuang 050000, China; 15632127751@163.com

**Keywords:** improved Tornado Optimizer with Coriolis force with Coriolis force, least squares support vector machine, bearing fault diagnosis, BAYES-ICEEMDAN-SNR

## Abstract

**Highlights:**

**What are the main findings?**

- An enhanced ICEEMDAN method integrating Bayesian optimization and adaptive signal-to-noise ratio (BAYES-ICEEMDAN-SNR) is proposed, which significantly improves the stability of vibration signal decomposition and the robustness of feature extraction.
- An improved Tornado Optimizer with Coriolis force (ITOC) is designed by incorporating Chebyshev chaotic mapping, Cauchy mutation, and dynamic opposition-based learning strategies, effectively enhancing global search capability and convergence accuracy.

**What are the implications of the main findings?**

- The constructed ITOC-LSSVM fault diagnosis model achieves a classification accuracy of 97.67% on the Case Western Reserve University bearing dataset, outperforming several comparative methods.
- This method provides an efficient and adaptive solution for intelligent fault diagnosis of rolling bearings under strong noise environments, demonstrating considerable potential for engineering applications.

**Abstract:**

To address the challenges of difficult feature extraction for rolling bearing vibration signals, low efficiency in optimizing diagnostic model parameters, and the tendency to get trapped in local optima, this paper proposes an improved ICEEMDAN feature extraction method based on Bayesian optimization and adaptive noise signal ratio enhancement (BAYES-ICEEMDAN-SNR) and combines it with the improved Coriolis force optimization algorithm (ITOC) to optimize the least squares support vector machine (LSSVM) fault diagnosis model. Firstly, Bayesian optimization is used to adaptively determine the noise parameters and introduce a dynamic signal-to-noise ratio adjustment mechanism to enhance the robustness of feature extraction; secondly, Chebyshev chaotic mapping, Cauchy mutation, and dynamic reverse learning strategies are applied to enhance the global search and local escape capabilities of ITOC, thereby optimizing the hyperparameters of LSSVM; and finally, the Keesey-Chestnut University bearing dataset and Huazhong University of Science and Technology gear dataset are used for verification. The experimental results show that the average fault identification accuracy of the proposed method reaches over 97%, which is superior to that of the comparison models, and the effectiveness of each core improvement module of the proposed model is verified through ablation experiments, providing an effective solution for intelligent fault diagnosis of rolling bearings and gears.

## 1. Introduction

As the core component of rotating machinery, rolling bearings directly affect the reliability, safety and service life of the whole equipment [1]. However, due to the complex operating conditions, strong noise interference and weak fault characteristics in the early stage, the bearing vibration signal often presents nonlinear, non-stationary and multi-scale complexity, which makes fault feature extraction and condition monitoring a major challenge. Therefore, the development of intelligent methods that can effectively suppress noise, adaptively extract robust features and achieve high-precision diagnosis has become a research hotspot in the field of condition monitoring and fault diagnosis.

In signal preprocessing and feature extraction, time-frequency domain analysis methods, such as Empirical Mode Decomposition (EMD) and its improved variants, have been widely used. Among them, the Improved Complete Ensemble Empirical Mode Decomposition with Adaptive Noise [2] (ICEEMDAN) with adaptive noise injection and residual calculation optimization strategy effectively suppresses mode aliasing and reduces reconstruction error. This method shows excellent denoising and feature separation capabilities in the fields of bearing fault diagnosis [3,4], marine rotating machinery, geological engineering and optical fiber sensing [5,6]. In order to further improve its adaptability, scholars have introduced various intelligent optimization methods to automatically optimize the key parameters of ICEEMDAN [7], such as Constrained Penalty Function Leech Algorithm (CPLA) [8], Crown Hedgehog Optimization Algorithm (CPO) [9,10], Improved Whale Optimization Algorithm (IWOA) [11,12] and Rimless Ice Optimization Algorithm (RIME) [13], which significantly improve the quality of signal decomposition and the robustness of feature extraction.

In the construction of a fault diagnosis model, Support Vector Machine (SVM) [14] and its variants are widely used because of their excellent performance in small sample and high-dimensional pattern recognition. Among them, Least Squares Support Vector Machine (LSSVM) [15] performs well in various fault diagnosis and state prediction tasks, such as bearing fault diagnosis [16], energy storage system risk assessment [17], pressure sensor temperature compensation [18], soil nutrient monitoring [19], shield tunnel settlement prediction [20] and runoff prediction [21]. However, the classification performance of LSSVM is highly dependent on its regularization and the selection of kernel function parameters. To solve this problem, a series of meta heuristic optimization algorithms have been introduced to automatically adjust these hyperparameters, such as the Modified Crown Hedgehog Optimization Algorithm (MICPO) [22], the improved artificial bee colony algorithm [23], Newton-Raphson Optimization (NRBO) [18], the Improved Wild Horse Optimization (IWHO) [24], and the Harris Hawk Optimization (HHO) [25]. Improved Sparrow Search Algorithm (ISSA) [26], Adaptive Mutation Particle Swarm Optimization (AMPSO) [27], Intelligent Black Kite Algorithm (IBKA) [21] and Improved Parrot Optimization Algorithm (IPO) [28] are algorithms that effectively improve the classification accuracy and generalization ability of the LSSVM model.

At the same time, deep learning models such as Convolutional Neural Networks (CNN), Long Short-Term Memory (LSTM) and Bidirectional LSTM show significant advantages in fault diagnosis due to their strong feature learning ability. These models are usually combined with optimization algorithms to optimize their structure or hyperparameters [29], such as Bayesian-optimized CNN-LSTM [30], Chaos Kepler Algorithm-optimized BiLSTM [31] and Improved Whale Algorithm-optimized BiLSTM [32]. In addition, in order to improve the interpretability of the model, some research has introduced interpretable feature mapping units or shap analysis to reveal the decision basis of the model. Multimodal information fusion is also an important trend to improve the robustness of diagnosis. By integrating signals from vibration, acoustics and other multiple sources, the equipment status can be evaluated more comprehensively [22].

Although the above research has made significant progress, there are still challenges in the rolling bearing fault diagnosis: (1) the existing ICEEMDAN method’s parameter adaptive mechanism is still not perfect, and its ability to extract weak fault features in strong noise background needs to be improved; (2) when optimizing classifiers such as LSSVM, most optimization algorithms still have the problem of premature convergence and are easy to fall into local optimization, which will affect the final performance of the model; and (3) at present, there is a lack of an end-to-end diagnosis framework that can simultaneously achieve high adaptive signal denoising, robust feature extraction and high-precision classification.

With the development of Industry 4.0, the running state of rolling bearings directly affects the reliability of the whole system. However, under actual working conditions, the early fault signals of bearings are often submerged in strong background noise. Existing layout technologies (such as VMD or traditional ICEEMDAN) often rely on manual parameter setting, and there are more problems of state mixing or reconstruction when processing non-stationary signals. In addition, the super parameter selection of a classifier (such as LSSVM) often determines the upper limit of recognition accuracy, so it is complex. A diagnostic framework that can deal with noise and optimize Bluetooth parameters is developed to achieve predictive maintenance with important engineering significance.

In view of the above challenges, this paper proposes a rolling bearing fault diagnosis method based on BAYES-ICEEMDAN-SNR-ITOC-LSSVM. Firstly, Bayesian optimization (BO) is introduced to adaptively determine the key parameters of ICEEMDAN, and the effective intrinsic mode function (IMF) components are selected by combining with the signal-to-noise ratio (SNR) criterion, so as to achieve high-quality noise reduction and feature enhancement of the signal under a strong noise background. Secondly, an improved tornado optimization algorithm (ITOC) is proposed, which optimizes the population initialization by the Chebyshev chaotic map, introduces the Cauchy mutation strategy to enhance the global exploration ability, and integrates a dynamic reverse learning strategy to avoid local optimization, so as to significantly improve the convergence accuracy and stability of the algorithm. Finally, the super parameters of LSSVM are adaptively optimized by using ITOC, and a high-performance fault classifier is constructed to realize the accurate recognition of bearing state. This paper aims to improve the quality of features through BAYES-ICEEMDAN-SNR, optimize the performance of the classifier through ITOC, and finally realize high-precision and robust diagnosis of bearing fault in a strong noise environment.

Although single components such as Bayesian optimization, chaotic mapping, Cauchy mutation and opposition learning are mature technologies in their respective fields, their collaborative integration into the specific application scenario of rolling bearing fault diagnosis constitutes the core innovation of this work. The selection of each component has a clear purpose and basis: Bayesian optimization provides a highly principled and sample-efficient hyperparameter optimization framework for the black box ICEEMDAN process, and its performance is better than grid search or manual parameter adjustment. Chebyshev chaotic map and Cauchy mutation were specially introduced to solve the recognized defects of the new TOC algorithm, namely, the lack of population diversity and premature convergence, and escape from local optimization by ensuring a more uniform search space and generating stronger disturbances. Dynamic opposition learning further optimizes this process. Importantly, the proposed adaptive SNR adjustment (BAYES-ICEEMDAN-SNR) within ICEEMDAN is not a simple additional function, but a targeted enhancement, which aims to solve the recognized weakness of the instability of the modal decomposition of standard ICEEMDAN under varying noise levels. Therefore, the innovation of this research is not to invent a new single algorithm, but to design a coherent and optimized diagnosis process, in which each improved component is aimed at a specific short plate in the traditional bearing diagnosis chain, and finally forms a proven robust adaptive system with better performance.

Therefore, this paper proposes a rolling bearing fault diagnosis method based on the integrated framework. The main contributions of this study are as follows:

1. An improved ICEEMDAN method based on Bayesian optimization and adaptive SNR adjustment (BAYES-ICEEMDAN-SNR) is proposed, which can adaptively determine the noise parameters and dynamically adjust the decomposition strategy to improve the robustness of feature extraction under strong noise.
2. An Improved Tornado Optimizer with Coriolis force (ITOC) is designed, which introduces Chebyshev chaotic map, Cauchy mutation and dynamic reverse learning mechanism to enhance the global search ability and convergence accuracy.
3. An end-to-end fault diagnosis framework is constructed, and the above improved method is applied to the parameter optimization of LSSVM. The classification accuracy of 97.67% is achieved on the CWRU bearing dataset and 97.41% on the HUST gear dataset, which are better than those of many comparison methods.
4. Experiments verify the adaptability and stability of the proposed method in a strong noise environment and provide a feasible scheme for intelligent fault diagnosis in industrial scenes.

The rest of this paper is organized as follows: the Section 2 introduces the proposed denoising method in detail. The open bearing dataset of Case Western Reserve University (CWRU) and the open gear dataset of Huazhong University of Science and Technology (HUST) are used to verify and compare the fault diagnosis performance of the proposed method. Finally, the Section 4 summarizes the full text.

## 2. Materials and Methods

### 2.1. Integrated Diagnostic Framework

**Figure 1:**
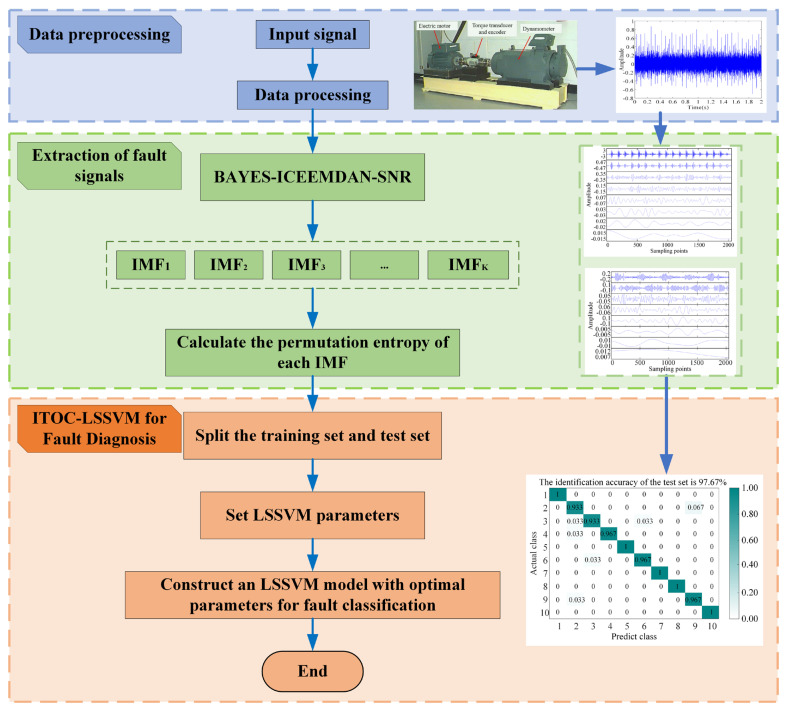
BAYES-ICEEMDAN-SNR -ITOC-LSSVM framework.

The proposed BAYES-ICEEMDAN-SNR-ITOC-LSSVM framework (Figure 1) synergizes adaptive signal processing with optimized machine learning. The process is streamlined into three core phases:

1. Adaptive Preprocessing: raw signals are decomposed via BAYES-ICEEMDAN-SNR to extract IMFs.
2. Feature Quantization: permutation entropy (PE) is calculated for the first eight IMFs to construct robust feature vectors.
3. Autonomous Classification: the ITOC algorithm fine-tunes LSSVM hyperparameters to perform final fault identification.

### 2.2. BAYES-ICEEMDAN-SNR

#### 2.2.1. Improved Complete Ensemble Empirical Mode Decomposition with Adaptive Noise

ICEEMDAN is an advanced adaptive signal decomposition method that has been further optimized based on CEEMDAN. This method effectively overcomes the modal mixing problem existing in traditional empirical mode decomposition and significantly reduces the reconstruction error and residual noise. The core improvement of this method is to adaptively introduce Gaussian white noise and suppress the mode mixing phenomenon by integrating multiple averaging steps. In addition, ICEEMDAN further optimizes the noise addition strategy and residual calculation logic, allowing for the extraction of purer intrinsic mode function components with clearer physical meanings.

The core principle of the ICEEMDAN algorithm is as follows [33]:

(1) Gaussian white noise $\omega^{i}$ is added to the original vibration signal f(t) to obtain

(1) $$f_{i}(t)=f(t)+\beta_{0}E_{1}\left(\omega^{i}\right)$$

where i represents the number of times Gaussian white noise is added, i=1,2,…,I. Using Empirical Mode Decomposition (EMD), the local mean of the i-th white noise component $f_{i}(t)$ is computed, and the average value is taken to obtain the first residual $r_{1}(t)$. At this point, the first IMF component is denoted as ${\mathrm{IMF}}_{1}=f(t)-r_{1}(t)$.

(2) $$r_{1}(t)=\frac{1}{l}\sum_{i=1}^{l}M\left[X^{\left(i\right)}\right]$$

(2) White noise $\omega^{\left(i\right)}$ is further added to the residual $r_{1}(t)$, resulting in the residual expression after the i-th addition of white noise:

(3) $$r_{1,i}(t)=r_{1}(t)+\beta_{1}E_{2}\left(\omega^{i}\right)$$

By using Formulas (3) and (4), the second IMF component is obtained, denoted as ${\mathrm{IMF}}_{2}=r_{1}(t)-r_{2}(t)$.

(4) $$r_{2}(t)=\frac{1}{l}\sum_{i=1}^{l}M\left\{\left[r_{1}(t)+\beta_{0}E_{2}\left(\omega^{i}\right)\right]\right\}$$

(3) According to Formula (5), the k-th IMF component is calculated.

(5) $${\mathrm{IMF}}_{k}=r_{k-1}(t)-r_{2}(t)$$

(6) $$r_{k}(t)=\frac{1}{N}\sum_{i=1}^{N}M\left[r_{k-1}(t)+\beta_{k-1}E_{k}\left(\omega^{i}\right)\right]$$

(4) The above steps are repeated until the residual R can no longer undergo EMD decomposition, resulting in the signal f(t):

(7) $$f(t)=\sum_{i=1}^{n}{\mathrm{IMF}}_{i}+r_{n}(t)$$

where n represents the total number of IMF components obtained through the decomposition.

#### 2.2.2. BAYES-ICEEMDAN-SNR Algorithm Principle

Although the ICEEMDAN algorithm effectively suppresses the modal mixing phenomenon existing in the traditional empirical mode decomposition, it still has certain limitations. A key issue is the deficiency of its adaptive noise selection mechanism. The selection of noise parameters has a significant impact on the quality of the decomposition results. However, in practical applications, the determination of noise parameters relies on experience or trial and error, lacking universally applicable criteria. To address this “adaptive noise selection problem”, an optimization strategy combining Bayesian optimization and adaptive signal-to-noise ratio (SNR) was proposed, which improved the noise selection process in ICEEMDAN, thereby enhancing the stability and accuracy of the decomposition results.

(1) Bayesian Optimization (BO)

Bayesian optimization [30] is a strategy for global optimization of complex functions, which can automatically select the noise parameters. Compared with traditional heuristic methods, this method has the advantages of high efficiency and fewer experimental iterations. Through Bayesian optimization, the automatic process of adaptive noise selection has been achieved, enabling more accurate selection of the most suitable noise parameters, thereby improving the stability of the decomposition results.

To implement the Bayesian optimization for SNR selection, a Matérn 5/2 kernel is employed for the Gaussian process (GP) to maintain a balance between smoothness and flexibility in modeling the objective function. The Expected Improvement (EI) is utilized as the acquisition function to identify the optimal SNR by balancing exploration and exploitation.

In the Bayesian optimization framework, Gaussian processes (GPs) are commonly used as surrogate models. Gaussian processes can model the objective function in a non-parametric manner, establishing a probabilistic relationship between the input and output based on the available observed data, and providing function value predictions and uncertainty estimates. Specifically, for a set of observation data $D={\left\{(x_{i},f(x_{i}))\right\}}_{i=1}^{t}$, a Gaussian Process can be described as:

(8) $$f(x)\sim GP(m(x),k(x,x^{\prime }))$$

In the ICEEMDAN algorithm, the standard deviation of the added noise (Nstd) is a key parameter affecting the decomposition performance. If Nstd is too small, it may lead to insufficient signal decomposition and residual mode mixing; if Nstd is too large, it may introduce spurious components or cause signal distortion. Traditional methods rely on the empirical setting of this parameter, lacking adaptive capabilities.

To address this, this paper introduces Bayesian optimization into ICEEMDAN to achieve automatic, global optimization of the Nstd parameter. Specifically, the decomposition process of ICEEMDAN is treated as a black-box function F(Nstd), whose output is an error metric for the decomposition result. The objective function is defined as the decomposition error, with common metrics such as the Mean Squared Error (*MSE*) between the original signal and the reconstructed signal:

(9) $$MSE=\frac{1}{N}\sum_{i=1}^{N}{\left(s_{i}-{\hat{s}}_{i}\right)}^{2}$$

(2) Adaptive Signal-to-Noise Ratio Optimization Strategy

Signal-to-noise ratio (SNR) is an important indicator that measures the strength of the signal’s effective components relative to the background noise intensity. Its mathematical definition is:

(10) $$SNR_{\mathrm{dB}}=10\;\log_{10}\left(\frac{P_{\mathrm{signal}}}{P_{\mathrm{noise}}}\right)$$

In the formula, $P_{\mathrm{signal}}$ and $P_{\mathrm{noise}}$ represent the power of the signal and noise, respectively. In the ICEEMDAN algorithm framework, the intensity of the auxiliary noise added (i.e., the standard deviation Nstd) directly affects the modal decomposition results: if Nstd is too small, it becomes difficult to effectively separate modes under high noise conditions; if Nstd is too large, it may introduce unnecessary distortion to high SNR signals, leading to over-decomposition or mode mixing. The traditional fixed Nstd parameter model lacks universality for signals in different SNR environments.

To address this issue, this paper proposes an adaptive signal-to-noise ratio (SNR) optimization strategy based on real-time signal quality. The core idea of this strategy is to dynamically adjust the strength of the injected noise according to the instantaneous SNR of the signal during the decomposition process, thus avoiding excessive interference when the signal is clear and providing sufficient decomposition driving force when the signal is polluted by noise. Specifically, an optimal noise standard deviation $Nstd_{opt}$ mapping function f(SNR) from the SNR value is established to achieve adaptive configuration of the parameter.

This method integrates SNR evaluation and noise adjustment mechanisms into the iterative loop of ICEEMDAN. The specific steps are as follows:

(a) At the start of each iteration to extract an Intrinsic Mode Function (IMF), the signal-to-noise ratio (SNR) estimate of the current residual signal $r_{k}(t)$ is calculated. To balance efficiency and practicality, a variance-based approximate estimation is used:

(11) $$\hat{SNR_{k}}=10\;\log_{10}\left(\frac{\mathrm{Var}(r_{k}(t))}{{\hat{\sigma }}_{\mathrm{noise}}^{2}}\right)$$

In the formula, ${\hat{\sigma }}_{\mathrm{noise}}^{2}$ can be obtained by estimating the background noise variance from the initial signal segment or prior knowledge.

(b) Design a monotonically decreasing mapping function $f({\hat{SNR}}_{k})$ to convert the estimated SNR value into a suggested noise strength coefficient $\beta_{k}$ for that iteration step. This function ensures that a low SNR corresponds to a higher noise enhancement coefficient, while a high SNR corresponds to a lower coefficient. A piecewise linear implementation can be defined as follows:

(12) $$\beta_{k}=\left\{\begin{array}{ccc} \beta_{\max}, & {\hat{\mathrm{SNR}}}_{k}<{\mathrm{Th}}_{\mathrm{low}} & \\ \beta_{\max}-\frac{\left(\beta_{\max}-\beta_{\min})({\hat{\mathrm{SNR}}}_{k}-{\mathrm{Th}}_{\mathrm{low}}\right)}{{\mathrm{Th}}_{\mathrm{high}}-{\mathrm{Th}}_{\mathrm{low}}}, & {\mathrm{Th}}_{\mathrm{low}}\leq {\hat{\mathrm{SNR}}}_{k}\leq {\mathrm{Th}}_{\mathrm{high}} & \\ \beta_{\min}, & {\hat{\mathrm{SNR}}}_{k}>{\mathrm{Th}}_{\mathrm{high}} & \end{array}\right.$$

where $\beta_{\min}$ and $\beta_{\max}$ represent the predefined upper and lower bounds of the noise strength coefficient, and ${\mathrm{Th}}_{\mathrm{low}}$ and ${\mathrm{Th}}_{\mathrm{high}}$ are the SNR thresholds that divide the signal quality intervals.

The basis for setting the SNR threshold: the piecewise linear mapping function in Formula (12) uses two SNR thresholds $Th_{high}$ and $Th_{low}$ to define high, medium and low signal quality regions. These thresholds are not arbitrarily set but determined based on the empirical observation of vibration signal characteristics and ICEEMDAN operation logic. High SNR indicates that the residual signal is relatively pure. At this time, injecting too strong noise may lead to unnecessary decomposition artifacts. Therefore, the minimum noise figure $\beta_{\min}$ is applied. On the contrary, low SNR means that the residual signal is dominated by noise, and stronger auxiliary noise is needed to effectively separate potential modes. The medium SNR region triggers linear interpolation to achieve a smooth transition.

The specific values of $Th_{high}$ and $Th_{low}$ initially refer to the common SNR range in bearing vibration analysis, and are then fine-tuned through preliminary experiments on the CWRU data subset to ensure that the adaptive mechanism can make an appropriate response under typical signal conditions. This design achieves a balance between the simplicity of the algorithm and the effective adaptive behavior.

(c) The generated baseline white noise $n_{k}(t)$ is normalized by its standard deviation, and then multiplied by the dynamic coefficient $\beta_{k}$ obtained from the mapping function and the globally optimized baseline strength $Ntsd^{*}$ (from the Bayesian optimization output in Section 2.1), forming the actual injected noise $N_{\mathrm{inj},k}(t)$ for that iteration step.

(13) $$N_{\mathrm{inj},k}(t)=Nstd^{*}\cdot \beta_{k}\cdot \frac{n_{k}(t)}{\mathrm{std}(n_{k}(t))}$$

The normalized noise exhibits stable unit variance properties, and when combined with the dynamic coefficient and the globally optimal parameter, it ensures the precision and consistency of the noise adjustment.

(d) The dynamically adjusted noise $N_{\mathrm{inj},k}(t)$ is added to the current residual signal, and the EMD decomposition is performed to extract the next IMF and update the residual signal. Steps (1) to (4) are repeated until the decomposition stopping criteria are met.

#### 2.2.3. BAYES-ICEEMDAN-SNR Procedure

**Figure 2:**
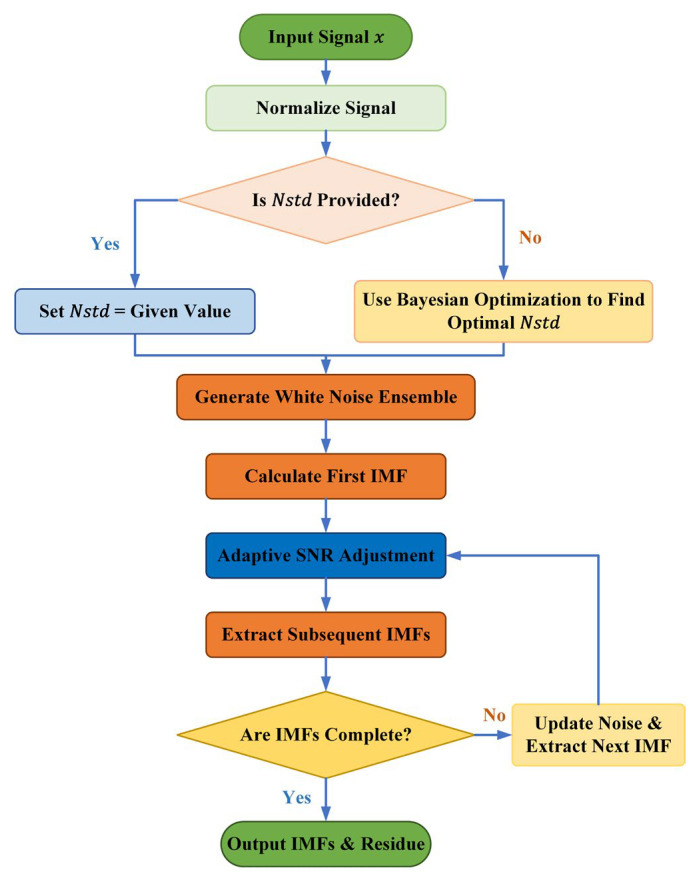
BAYES-ICEEMDAN-SNR Algorithm Flowchart.

The flowchart of the proposed BAYES-ICEEMDAN-SNR method is shown in Figure 2. The specific steps are as follows:

**Step 1**: Input the signal to be decomposed, x, and related parameters, and normalize the signal so that its mean is zero and the unit standard deviation is zero.

**Step 2**: If *Nstd* is not provided or is NaN, start the Bayesian optimization. The objective function calculates the decomposition error under different *Nstd* values and searches for the optimal *Nstd* within the range of [0.1, 1.0]. If *Nstd* is provided, use its value directly.

**Step 3**: Generate NR groups of white noise sequences with the same length as the signal. Perform EMD decomposition on each white noise sequence to obtain its intrinsic mode function set (IMF), which is used for subsequent noise injection.

**Step 4:** Normalize the first IMF of each white noise sequence by standard deviation, multiply it by the optimized *Nstd*, and add it to the normalized signal. Then, perform EMD decomposition on each noise-enhanced signal to extract the first IMF. Calculate the average residual of all NR decompositions to obtain the first IMF.

**Step 5**: For each subsequent IMF extraction step, adjust the normalization of the injected noise according to SNRFlag. Use the standard deviation of the current residual to dynamically scale the noise, achieving adaptive signal-to-noise ratio (SNR) control. If there are insufficient white noise IMFs, directly perform EMD decomposition on the current residual. Repeat this process until no new IMF can be extracted from the residuals.

**Step 6:** Restore all intrinsic mode functions and the final residual to the original signal amplitude and output the complete set of intrinsic mode functions and the iterative records of each EMD decomposition.

### 2.3. Permutation Entropy (PE)

Permutation entropy is an indicator for measuring the complexity of time series. By using embedding dimension and time-delay reconstruction of sub-sequences, the sub-sequences are sorted level by level based on the idea of stepwise variation and mapped to the permutation space. Shannon entropy is calculated to evaluate the complexity. The magnitude of permutation entropy is inversely proportional to the regularity of the time series.

The calculation of permutation entropy is as follows:

(1) In a time series of length N, denoted as u(1), u(2), u(3), …, u(N), an embedding dimension m and a time delay L are specified. The original sequence is reconstructed, and each subsequence is represented as X(i).

(14) X(i)=u(i),u(i+L),…,u(i+(m−1)L)

(2) For each subsequence X(i), perform an increasing order sorting within it, i.e., if two values are equal, sort them according to their indices I. This way, each M-dimensional subsequence X is mapped to one of K possible permutations.
(3) After the above step, represent the continuous M-dimensional subsequences by a symbol sequence, where the number of symbols is G. The probability distribution of all symbols is represented as P, and the entropy is calculated as H. The permutation entropy of the time series U is then given by:

(15) $$H(m)=-\sum_{j=1}^{K}P_{j}\ln P_{j}$$

where $p_{j}$ is the probability of the i-th symbol (permutation) occurring, and K is the total number of possible symbols. This entropy value quantifies the complexity of the time series, with higher entropy indicating greater complexity and lower entropy indicating more regularity in the sequence.

### 2.4. Improved Tornado Optimizer with Coriolis Force with Coriolis Force (ITOC)

#### 2.4.1. Tornado Optimizer with Coriolis Force with Coriolis Force (TOC)

Tornado Optimizer with Coriolis force with Coriolis force (TOC) is a new nature-inspired heuristic algorithm. Its core idea is to simulate the formation and evolution process of a tornado. The algorithm consists of three roles, storm, thunderstorm, and tornadoes, which move and interact in the search space and eventually find the global optimal solution. The core principle and mechanism of this algorithm are as follows [34]:

(1) Population Initialization and Classification

The initialization process of the TOC algorithm involves randomly generating the positions of a set of design variables (storms, thunderstorms, and tornadoes). First, the algorithm randomly generates *n* individuals in the search space. These individuals are divided into three categories to simulate the different stages of tornado formation:

1. Tornadoes ($n_{o}$): Typically set to 1, representing the current best solution found.
2. Thunderstorms ($n_{t}$): A portion of the relatively better individuals, which are potential excellent solutions.
3. Windstorms ($n_{w}$): The remaining ordinary individuals in the population, which are the main force for exploring the search space.

Their relationships are as follows:

(16) $$n=n_{t}+n_{o}+n_{w}$$

The initialization formula is:

(17) $$y_{i,j}=l_{j}+rand\times \left(u_{j}-l_{j}\right)$$

where $y_{i,j}$ is the initial position of the i-th individual in the j-th dimension. $u_{j}$ and $l_{j}$ are the lower and upper bounds for the j-th dimension, respectively. rand is a random number between 0 and 1.

(2) Evolution and Movement of Windstorms

The storms are the main executors of behavior detection in algorithms. Their movements and evolution directions are influenced by tornadoes (the best solution) and thunderstorms (the suboptimal solution). The mathematical model of the evolution process is as follows:

1. Speed Update of Windstorms

The speed update of windstorms takes into account the influence of the Coriolis force. The update formula is:

(18) $${\vec{v}}_{t+1}^{i}=\left\{\begin{array}{cc} \eta \left(\mu {\vec{v}}_{t}^{i}-c\frac{f\times R_{l}}{2}+\sqrt{CF_{l}}\right) & \mathrm{if}\;rand\geq 0.5\\ \eta \left(\mu {\vec{v}}_{t}^{i}-c\frac{f\times R_{r}}{2}+\sqrt{CF_{r}}\right) & \mathrm{if}\;rand<0.5 \end{array}\right.$$

where ${\vec{v}}_{t+1}^{i}$ is the speed of the i-th windstorm at the t+1-th iteration. ${\vec{v}}_{t}^{i}$ is the speed of the i-th windstorm at the t-th iteration. f is the Coriolis parameter. η is the contraction factor, which controls the convergence speed of the windstorm. μ is the fuzzy adaptive kinetic energy factor. $R_{l}$ and $R_{r}$ are the radii of curvature for the northern and southern hemispheres, respectively. c is the random factor.

(19) f=2Ωsin(ϕ)

where Ω is the Earth’s angular velocity of rotation. ϕ is the latitude.

(20) $$\eta =\frac{2}{\sqrt{2-\chi -\frac{\chi^{2}}{4}}}$$

where χ is the acceleration factor, with a value of 4.10.

(21) $$\mu =0.5+\frac{rand}{2}$$

(22) $$R_{l}=\frac{2}{1+e^{-(t-T)/2}}$$

(23) $$R_{r}=-\frac{2}{1+e^{-(t-T)/2}}$$

where t is the current iteration number. T is the maximum number of iterations.

(24) $$c=b_{r}\times \delta_{1}\times wr$$

where $b_{r}$ is a constant, with a value of 100,000. $\delta_{1}$ is the symbol change factor. CF is the Coriolis force.

2 Windstorm Position Update

The windstorms update their positions based on their updated speed, learning from the tornado or thunderstorms, evolving into either a tornado or a thunderstorm.

(a) Evolving into a Tornado:

(25) $${\vec{y}}_{w_{i}}^{t+1}={\vec{y}}_{w_{i}}^{t}+2\times \alpha \times ({\vec{y}}_{o_{i}}^{t}-rand_{w})+{\vec{v}}_{i}^{t+1}$$

where ${\vec{y}}_{w_{i}}^{t+1}$ and ${\vec{y}}_{w_{i}}^{t}$ are the new position vector and current position vector of the windstorm at the t+1-th and t-th iterations, respectively. ${\vec{y}}_{o_{i}}^{t}$ is the current position vector of the i-th tornado at the t-th iteration. ${\vec{y}}_{o_{i}}^{t}-rand_{w}$ represents the difference between the evolution of the windstorm towards the tornado and the random formation of the windstorm. $rand_{w}$ and α are random values, with specific definitions given in Formulas (26) and (27).

(26) $$rand_{w}={\vec{y}}_{w_{i}}\left(\left\lfloor n_{w}\times rand\left(1,n_{w}\right)\right\rfloor +1\right)$$

where $rand_{w}$ is the index vector used for randomly selecting windstorms.

(27) $$\alpha =\left|2a_{y}\cdot rand-rand\right|$$

where rand is a random value generated within the range of [0,1], following a uniform distribution. $a_{y}$ represents an exponential parameter, with the value given by:

(28) $$a_{y}=\frac{\left(T-\left(t^{a_{0}}/T\right)\right)}{T}$$

where $a_{0}$ is a constant, with a value of 2.0.

(b) Evolving into a Thunderstorm

(29) $${\vec{y}}_{w_{j}+\overset{n_{{\dot{w}}_{k}}}{\underset{1}{\sum }}}^{t+1}={\vec{y}}_{w_{j}+\overset{n_{{\dot{w}}_{k}}}{\underset{1}{\sum }}}^{t}+2\times rand\times \left({\vec{y}}_{t_{i}}^{t}-{\vec{y}}_{w_{j}+\overset{n_{{\dot{w}}_{k}}}{\underset{1}{\sum }}}^{t}\right)+2\times rand\times \left({\vec{y}}_{o_{i}}^{t}-{\vec{y}}_{w_{j}+\overset{n_{{\dot{w}}_{k}}}{\underset{1}{\sum }}}^{t}\right)$$

where ${\vec{y}}_{w_{j}+\overset{n_{{\dot{w}}_{k}}}{\underset{1}{\sum }}}^{t+1}$ is the new position vector of the windstorm evolving into a thunderstorm at the t+1-th iteration. ${\vec{y}}_{w_{j}+\overset{n_{{\dot{w}}_{k}}}{\underset{1}{\sum }}}^{t}$ is the current position vector of the windstorm evolving into a thunderstorm at the t-th iteration. ${\vec{y}}_{t_{i}}^{t}$ is the current position vector of the i-th thunderstorm at the t-th iteration.

(3) Evolution of Thunderstorms

Thunderstorms also evolve towards the tornado (best solution), further enhancing the algorithm’s local exploitation capability. The position update formula for thunderstorms is as follows:

(30) $${\vec{y}}_{t_{i}}^{t+1}={\vec{y}}_{t_{i}}^{t}+2\times \alpha \times \left({\vec{y}}_{t_{i}}^{t}-y_{o_{\zeta }}^{t}\right)+2\times \alpha \times \left({\vec{y}}_{t_{\vec{p}}}^{t}-{\vec{y}}_{t_{i}}^{t}\right)$$

where ${\vec{y}}_{t_{i}}^{t+1}$ and ${\vec{y}}_{t_{i}}^{t}$ are the new position vector and current position vector of the thunderstorm evolving into a tornado at the t+1-th and t-th iterations, respectively. $y_{o_{\zeta }}^{t}$ is the position vector of the tornado at the random index ζ. ${\vec{y}}_{t_{\vec{p}}}^{t}$ is the position vector of the thunderstorm at the random index vector $\vec{p}$, which is used to randomly select the thunderstorm. Its specific definition is given in Formula (25).

(31) $$\vec{p}=\left\lfloor n_{t}\cdot rand\left(1,n_{t}\right)+1\right\rfloor$$

(4) Random Formation of Windstorms

To enhance global exploration ability and avoid local optima, the algorithm simulates the random formation process of windstorms. When windstorms or thunderstorms are very close to the tornado, i.e., the distance is smaller than a threshold ν, they will be reinitialized at random positions. The random formation formula is:

(32) $${\vec{y}}_{w_{i}}^{t+1}={\vec{y}}_{w_{i}}^{t}-\left(2\times a_{y}\times (rand\times (l-u)-l)\right)\times \delta_{2}$$

where $a_{y}$ is an adaptive parameter that decreases with the number of iterations. l and u are the lower and upper bounds of the search space, respectively. $\delta_{2}$ is the symbol change function.

#### 2.4.2. Improvements to the Tornado Optimizer with Coriolis Force with Coriolis Force

**Figure 3:**
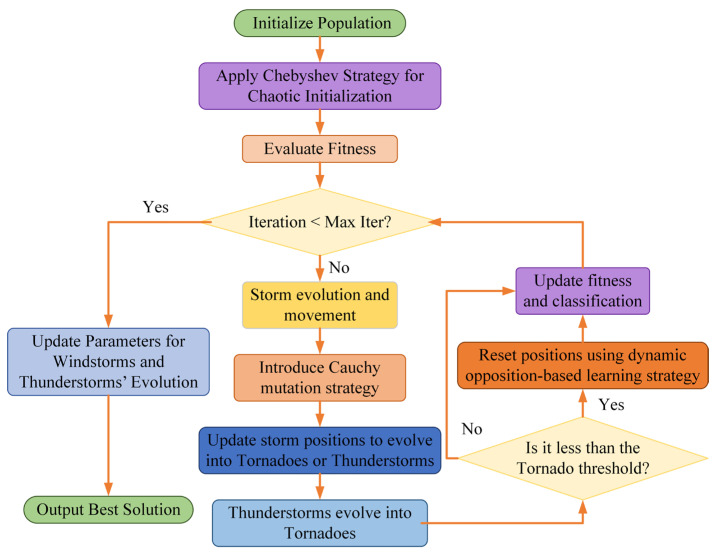
Optimization process of the ITOC method.

This paper presents an improved TOC optimization algorithm, which enhances its performance by incorporating three strategies (Chebyshev chaotic mapping, Cauchy mutation, and dynamic reverse learning). The improved algorithm is named ITOC, and its specific process is shown in Figure 3.

(1) Chebyshev Chaotic Mapping Strategy

During the initialization stage, the TOC algorithm generates the population through uniform random distribution, which results in the population not being able to fully cover the search space. The Chebyshev chaotic mapping can produce a more uniform and thorough initial population, ensuring a wider distribution of individuals and thereby enhancing the global search capability. Therefore, the Chebyshev chaotic mapping strategy is adopted to improve the TOC algorithm, replacing the random initialization step and providing a better starting point for the subsequent evolution.

The Chebyshev chaotic mapping is a chaotic system based on Chebyshev polynomials, and its mapping formula is [35]:

(33) $$x_{n+1}=\cos(\lambda \cdot \cos^{-1}x_{n})$$

where λ is an integer parameter, $x_{n}\in [-1,1]$.

(2) Cauchy Mutation Strategy

To address the issues of local optimum trapping and premature convergence in the original TOC algorithm, the Cauchy mutation strategy was introduced in the storm position update stage of the original algorithm. The heavy-tailed characteristic of this strategy provides a powerful perturbation mechanism, effectively solving the problems of local optimum and premature convergence, while retaining the original physical inspiration advantage of the TOC algorithm. This significantly improves the global search ability and convergence accuracy. The formula of the Cauchy mutation strategy is as follows [36]:

(34) Cauchy(0,g)=g×tan(p×(rand−0.5))

In the formula: g is the scale parameter, which controls the mutation intensity. Cauchy(0,g) is the Cauchy random number.

After introducing the Cauchy mutation strategy, the windstorm position update formula is:

1 Evolving into a Tornado:

(35) $${\vec{y}}_{w_{i}}^{t+1}={\vec{y}}_{w_{i}}^{t}+2\times \alpha \times ({\vec{y}}_{o_{i}}^{t}-rand_{w})+{\vec{v}}_{i}^{t+1}+\eta \cdot Cauchy(0,\gamma )$$

2 Evolving into a Thunderstorm:

(36) $${\vec{y}}_{t_{i}}^{t+1}={\vec{y}}_{t_{i}}^{t}+2\times \alpha \times ({\vec{y}}_{t_{i}}^{t}-y_{o_{\zeta }}^{t})+2\times \alpha \times ({\vec{y}}_{t_{p}}^{t}-{\vec{y}}_{t_{i}}^{t})+\eta \cdot Cauchy(0,\gamma )$$

(3) Dynamic Reverse Learning Strategy

In order to improve the reset efficiency of the TOC algorithm and accelerate the speed of escaping from local optimal solutions, a dynamic reverse learning strategy is introduced during the random formation stage of the storm. This strategy generates reverse solutions for the current solution, providing direction perturbation for the reset process. The mathematical model of the dynamic reverse learning strategy is as follows [37]:

(37) $$\tilde{x}=x+r_{1}\cdot \left(r_{2}\cdot \left(U_{b}+L_{b}-x\right)-x\right)$$

where $\tilde{x}$ is the reverse solution. $r_{1}$ and $r_{2}$ are random numbers between [0, 1]. x is the current solution.

When a windstorm or thunderstorm is marked for random reset, the dynamic reverse learning strategy generates the reset position as follows:

(38) $${\vec{y}}_{new}=\vec{u}b+\vec{l}b-{\vec{y}}_{old}+\delta \cdot rand$$

where δ is the adaptive perturbation intensity.

#### 2.4.3. Improvements to the Tornado Optimizer with Coriolis Force: Algorithm Flow

The execution flowchart of the proposed improved TOC algorithm is shown in Figure 3. The specific steps of the optimization algorithm are as follows:

**Step 1:** Use the Chebyshev chaotic mapping to generate the initial population, evaluate the fitness value of each individual, and sort the population according to the fitness value to divide it into three categories: tornado (the best individual), thunderstorm (the suboptimal individual), and storm (the remaining individuals).

**Step 2:** In each iteration, update the speed and position of the storm according to the adaptive parameters, allowing them to develop towards the tornado and thunderstorm directions. To enhance the global search ability of the algorithm, the Cauchy mutation strategy is introduced.

**Step 3:** The thunderstorm individuals update their positions by moving towards a randomly selected tornado position and the current global best position, thereby strengthening local utilization. During this process, a certain probability of fusion mutation is performed to avoid getting stuck in local optima.

**Step 4:** If the learning strategy based on dynamic opposition at the tail is enabled, perform mutation operations on the three population categories of tornado, thunderstorm, and storm, further optimizing the individual positions and improving the quality and diversity of the solutions.

**Step 5:** If the fitness of the current best solution is lower than the set tornado threshold, trigger the position reset mechanism and regenerate some individuals using the dynamic opposition learning strategy. Repeat steps 2 to 4 until the maximum number of iterations is reached.

**Step 6:** Output the global optimal solution and its fitness value, and record the best fitness of each generation.

### 2.5. Least Squares Support Vector Machine (LSSVM)

Support Vector Machine (SVM) is a widely used machine learning method, especially effective in handling classification problems. However, it faces efficiency issues when dealing with large-scale datasets. The Least Squares Support Vector Machine (LSSVM) solves this problem by introducing Lagrange multipliers to transform the original problem into a dual problem. Finally, the optimal classification hyperplane is obtained by solving a set of linear equations. Compared with traditional Support Vector Machines, this method simplifies the solution process while maintaining high solution efficiency, especially when dealing with complex nonlinear problems.

Let there be m samples, each of n dimensions:$\left\{x_{k},y_{k}\right\}$, $y_{k}\in \left\{-1,1\right\}$$x_{k}\in R^{n}$, k=1,2,3…m. The objective function and inequality constraints of the SVM algorithm are as follows:

(39) $$\min_{W,b,\zeta }J_{P}(W,\zeta )=\frac{1}{2}W^{T}W+c\sum_{k=1}^{m}\zeta_{k}$$

(40) $$s.t.\;y_{k}⩾W^{T}\varphi \left(x_{k}\right)+b+\zeta_{k}$$

where W is the normal vector of the hyperplane, ζ is the prediction error value, $\varphi \left(x_{k}\right)$ is the kernel function, c is the penalty factor, and b is a constant.

The LSSVM algorithm transforms Equations (39) and (40) into the following equations to improve the speed of solving the problem.

(41) $$\min_{W,b,e}J_{P}(W,e)=\frac{1}{2}W^{T}W+\frac{\gamma }{2}\sum_{k=1}^{m}e_{k}$$

(42) $$s.t.\;y_{k}=W^{T}K\left(x_{k},x\right)+b+e_{k}$$

where γ is the penalty factor for the prediction error, and e is the square of the SVM prediction error ζ, i.e., $e=\zeta^{2}$.

By constructing the Lagrange transformation polynomial, the following transformation is performed:

(43) $$L(W,b,e,\alpha )=J_{P}(W,e)-\sum_{k=1}^{m}\alpha_{k}\left[W^{T}K\left(x_{k},x\right)+b+e_{k}-y_{k}\right]$$

To obtain the linear equations, we take the partial derivatives of W, b, $e_{k}$ and $\alpha_{k}$ in Equation (37) and set them equal to zero. Solving this system of equations gives the calculation formula of the LSSVM algorithm, as shown in Equation (38).

(44) $$y(x)=\sum_{k=1}^{m}\alpha_{k}K\left(x_{k},x\right)+b$$

where $K\left(x_{k},x\right)$ is the kernel function.

(45) $$K=\exp\left(-\frac{\left\|x_{k}-x\right\|}{2\sigma^{2}}\right)$$

where $\sigma^{2}$ represents the parameter of the RBF kernel function.

## 3. Experimental Verification

### 3.1. Improving the Performance Verification of the Tornado Algorithm

This paper conducted a systematic simulation experiment in the MATLAB R2023b environment to rigorously verify the performance of the proposed optimization algorithm. The experimental parameter configuration is as follows: population size N = 30, and maximum number of iterations T = 100. As shown in Table 1, 12 standard test functions were selected as evaluation benchmarks, among which F1–F4 are unimodal functions, F5–F9 are multimodal functions, and F10–F12 are fixed-dimensional functions.

**Figure 4:**
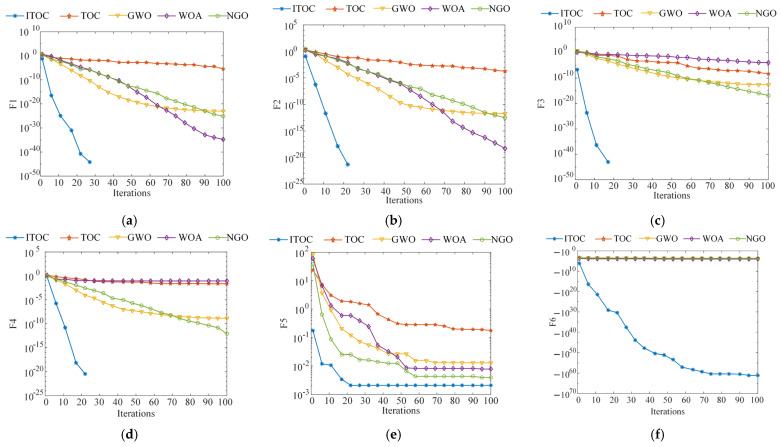

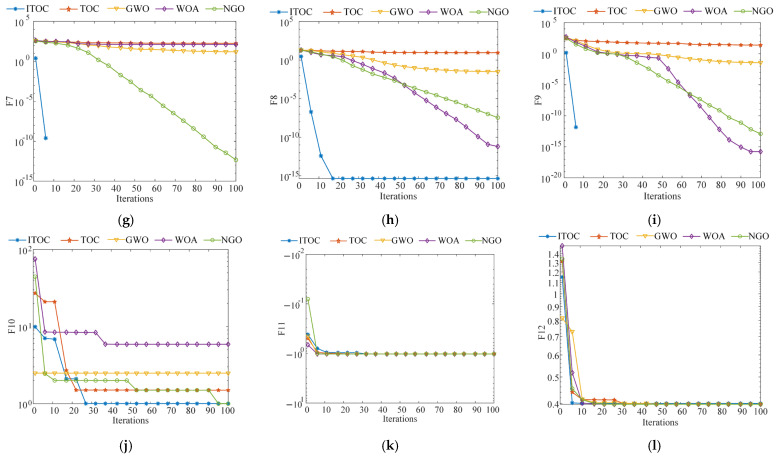
Test Function Run Results, (**a**) F1 function test results plot, (**b**) F2 function test results plot, (**c**) F3 function test results plot, (**d**) F4 function test results plot, (**e**) F5 function test results plot, (**f**) F6 function test results plot, (**g**) F7 function test results plot (**h**) F8 function test results plot, (**i**) F9 function test results plot, (**j**) F10 function test results plot, (**k**) F11 function test results plot, (**l**) F12 function test results plot.

To further verify the effectiveness and robustness of the algorithm, this paper designs a comparative experiment. Four representative meta-heuristic algorithms are selected as the control group, namely the original tornado optimization algorithm (TOC), the grey wolf optimization algorithm (GWO), the whale optimization algorithm (WOA), and the northern bald ibis optimization algorithm (NGO). The test results of the functions are shown in Table 2 and Figure 4.

From the test function results in Table 2 and the convergence curves in Figure 4, it can be seen that when testing the unimodal functions F1–F4, the proposed algorithm consistently converges to the theoretical optimal value of 0, demonstrating excellent stability. Its convergence speed is significantly faster than that of GWO, WOA, NGO, and the original TOC algorithm. This indicates that the algorithm has high convergence accuracy and search efficiency when solving optimization problems with clear gradient information. In the tests of multimodal functions F5–F9, this algorithm not only quickly finds the global optimal solution but also has good stability. Compared with the original TOC algorithm, this algorithm has a stronger ability to escape from local optima and stronger robustness. When dealing with complex optimization problems such as high-dimensional fixed-dimensional functions F10–F12, this algorithm maintains excellent performance. The ability to avoid local optima and the convergence speed of this algorithm are both superior to those of the comparison algorithms, further verifying its effectiveness and practicality in handling high-dimensional multi-peak optimization problems.

### 3.2. Validation of CWRU Bearing Experimental Signals

#### 3.2.1. Bearing Experimental Parameters and Vibration Signal Data Preprocessing

**Figure 5:**
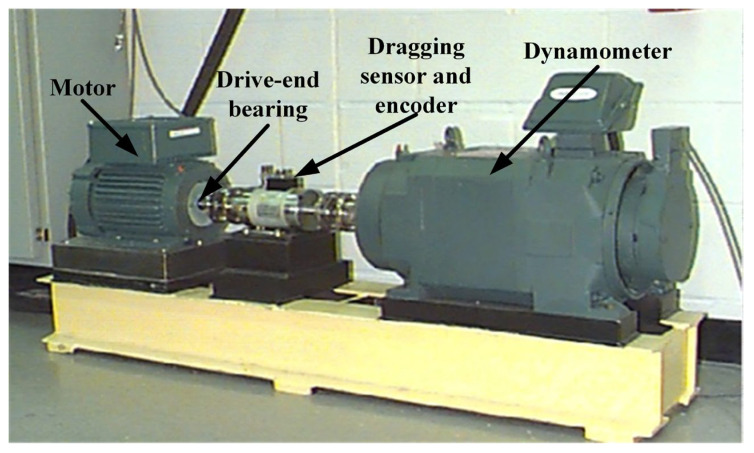
Rolling bearing failure test bench.

This study utilized the actual bearing data from Case Western Reserve University (CWRU) to test the fault diagnosis capability of the proposed method [38]. The rolling bearing fault simulation test bench, as shown in Figure 5, consists of four main components: a motor, a torque sensor, a power meter, and an electronic controller. This study selects data from the drive-end bearing, with a bearing model of SKF6205-2RS, a sampling frequency of 12 kHz, and a rotational speed of 1797 RPM. The structural parameters of the bearing are detailed in Table 3.

The experimental data include 10 different bearing health states, including one normal (fault-free) state and three typical failure types: inner ring failure, rolling element failure, and outer ring failure. Each failure type includes defect diameters of 0.007 inches, 0.014 inches, and 0.021 inches.

**Figure 6:**
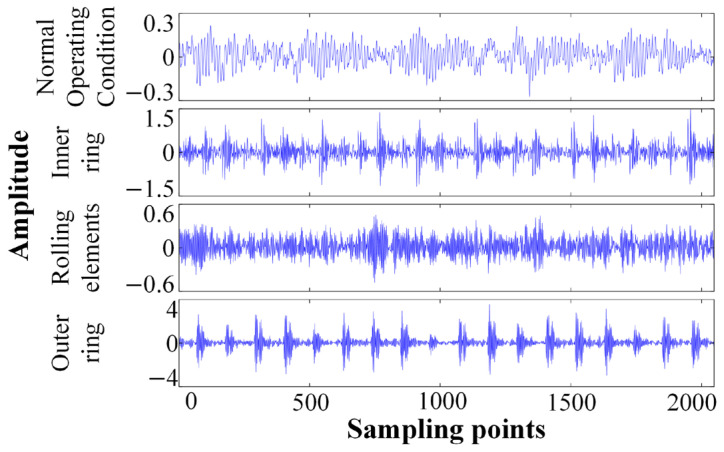
Vibration signal time-domain diagram.

The vibration signals were sampled using the sliding window method to generate a dataset for model training. The sliding window length was set to 1000 data points, and each sample contained 2048 consecutive vibration data points. Also, 120 independent samples were extracted for each bearing state. Therefore, for each rotational speed condition, a dataset containing 1200 samples was obtained, with each sample having a dimension of 1 × 2048. The result samples were randomly divided into a training set and a test set in a 7:3 ratio. Taking a group of samples of ten states as an example, the time-domain analysis of the vibration signals is shown in Figure 6.

#### 3.2.2. Bearing Fault Feature Extraction

This study decomposed the original vibration signal using ICEEMDAN to obtain a series of IMF components. In the selection of IMF components, the influence of local defects in the bearing was considered. When the bearing was under local defect conditions, the fault excitation caused high-frequency resonances, and the impact components were mainly distributed in the medium and high-frequency segments of the signal. The first few IMF components obtained through decomposition usually contain the most abundant fault influence information and resonance frequency band characteristics, while the subsequent components often contain low-frequency trend terms and background noise interference. On this basis, the first eight IMF components were selected as the basis for subsequent feature extraction.

When constructing the feature vector, entropy features, as indicators that can effectively quantify the complexity and irregularity of the signal, are widely used in mechanical fault diagnosis. Common entropy features include permutation entropy (PE), sample entropy (SE), and energy entropy (EE). Energy entropy is simple to calculate and can reflect the distribution of signal energy in different frequency bands, but has low sensitivity to fault impact characteristics. Sample entropy has advantages in measuring the complexity of time series, but has a high computational cost, is more sensitive to data length and parameter selection, and has poor stability. In contrast, permutation entropy has unique advantages: it has strong anti-noise and anti-interference capabilities, is particularly sensitive to subtle changes in dynamic time series, and is very suitable for characterizing the microscopic signal changes caused by bearing faults; its calculation efficiency is high, has clear physical meaning, can effectively measure the randomness and complexity of time series; and for the common nonlinear and non-stationary characteristics of bearing fault signals, permutation entropy can provide robust feature representations. Based on the above analysis, permutation entropy was selected to construct the feature vector.

**Figure 7:**
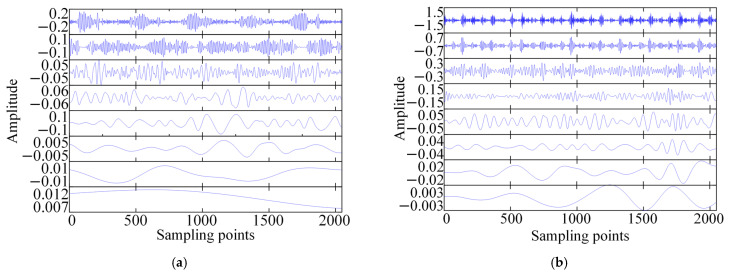

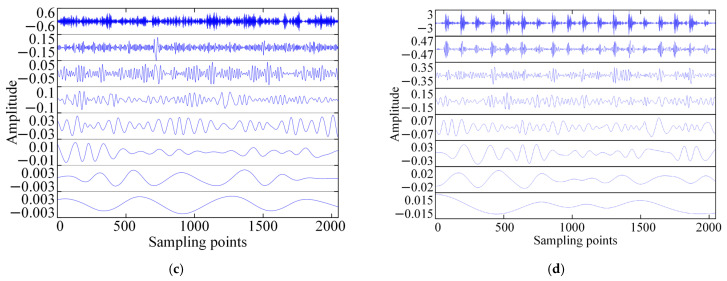
IMF components of the bearing under various states, (**a**) IMF in normal condition, (**b**) inner ring failure of bearing IMF, (**c**) bearing rolling element failure IMF, and (**d**) outer ring failure of bearing IMF.

The permutation entropy values of the selected first eight IMF components were calculated to form an 8-dimensional feature vector, which comprehensively represents the differences in dynamic characteristics of the bearing under different working conditions. The IMF components of each bearing state obtained through ICEEMDAN decomposition are shown in Figure 7. The *Nstd* value, SNRFlag and other parameter values of ICEEMDAN optimized by Bayesian optimization and adaptive SNR optimization strategy are shown in Table 4.

#### 3.2.3. Bearing Fault Identification

**Figure 8:**
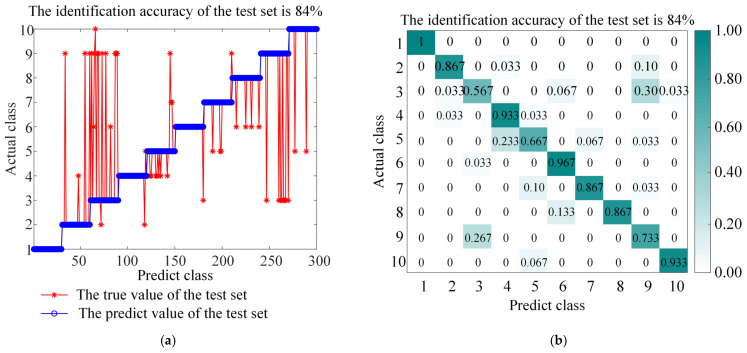
Accuracy and confusion matrix for LSSVM, (**a**) LSSVM classification accuracy, and (**b**) LSSVM confusion matrix.

**Figure 9:**
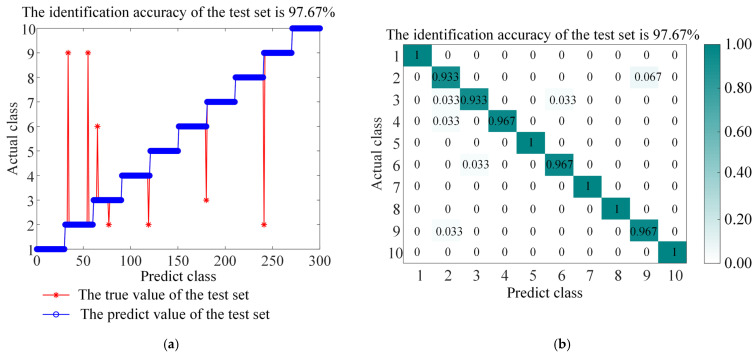
Accuracy and confusion matrix for BAYES-ICEEMDAN-SNR-ITOC-LSSVM, (**a**) BAYES-ICEEMDAN-SNR-ITOC-LSSVM classification accuracy, and (**b**) BAYES-ICEEMDAN-SNR-ITOC-LSSVM confusion matrix.

The ITOC optimization algorithm was used to optimize the regularization parameters of LSSVM and the parameters of the RBF kernel function. The test dataset was used for fault diagnosis, and the performance was compared. To verify the superiority and effectiveness of this method, comparative experiments were conducted between ITOC-LSSVM and LSSVM, TRP-SVD-SVM, RP-SVD-SVM, WEST-ICNN-IHBA-LSSVM, and EMD-INGO-LSSVM. The diagnostic results of LSSVM and ITOC-LSSVM are shown in Figure 8 and Figure 9 respectively, while the results of other models are presented in Table 5. The experimental results indicate that the recognition rate of this method reaches 97.67%, and the classification accuracy is superior to that of other methods, proving the superiority of this method in fault diagnosis. To account for the stochastic nature of the metaheuristic optimization, the proposed ITOC-LSSVM was executed 20 times independently. The average accuracy is reported as 97.67% ± 0.32% (95% Confidence Interval).

### 3.3. Gear Fault Diagnosis Test Verification

#### 3.3.1. Test Data Description and Pretreatment

**Figure 10:**
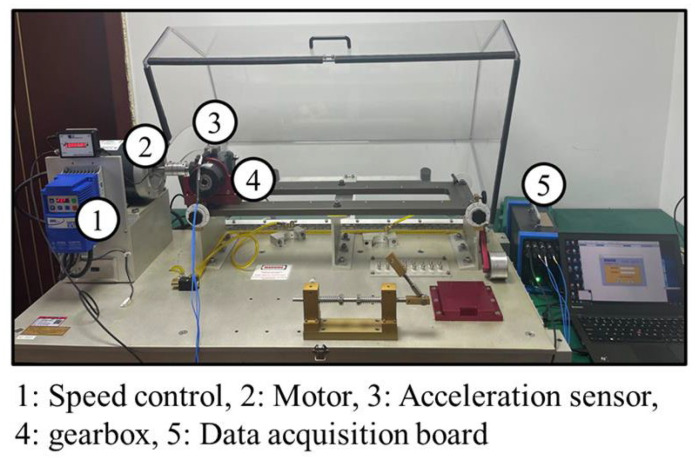
Test rig of HUST gearbox dataset.

In order to further verify the generalization ability of the proposed method on various mechanical components, a second experiment was conducted using the gearbox dataset published by Huazhong University of Science and Technology (HUST) [39]. The test bench mainly includes a speed control unit, motor, acceleration sensor, gearbox and data acquisition board, as shown in Figure 10. The Hub City M2 gearbox with a transmission ratio of 1.5:1 was used in the test.

**Figure 11:**
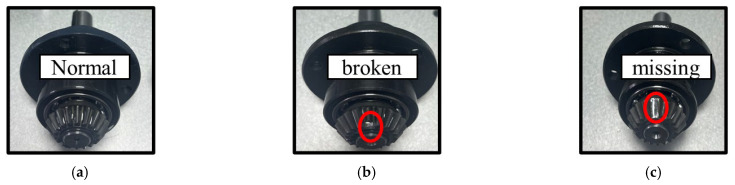
The gearbox in three health states, (**a**) normal, (**b**) broken tooth, and (**c**) missing tooth.

This experiment includes three typical health states of gears, normal, broken tooth and missing, as shown in Figure 11. In order to simulate complex actual working conditions, the experiment was conducted at four speeds from 20 Hz to 35 Hz and four corresponding loads, as shown in Table 6. The sampling frequency of data acquisition is set to 25.6 kHz.

In data processing, the original vibration signal is sliced by a sliding window. The sliding window span is set as 1000 sampling points; each sample contains 2048 continuous vibration data points, 120 groups of samples are extracted from each state, and a total of 1080 test samples are obtained. Then, the dataset was randomly divided into a training set and a test set according to the ratio of 3:1, in which the training samples were 90 groups, and the test samples were 30 groups.

#### 3.3.2. Fault Feature Extraction and Recognition

The feature extraction process is the same as that in Section 3.2.2. Firstly, the BAYES-ICEEMDAN-SNR proposed in this paper is used to adaptively decompose the gear vibration signal. In the decomposition process, taking into account the distribution characteristics of gear meshing frequency and its multiple frequency components, this paper extracts the first eight IMF components containing the main fault feature information. For each order component, the normalized permutation entropy (PE) value is calculated, and an 8-dimensional feature vector that can characterize the nonlinear dynamic characteristics of the gear is constructed.

In the fault classification stage, the improved tornado optimization algorithm (ITOC) is used to globally optimize the regularization parameter γ and kernel function parameter $\sigma^{2}$ of LSSVM. The improved tornado optimization algorithm (ITOC) can effectively avoid the optimization process falling into local optimization by simulating the evolution mechanism of tornadoes, so as to obtain more robust LSSVM model parameters.

#### 3.3.3. Result Analysis and Conclusion

**Figure 12:**
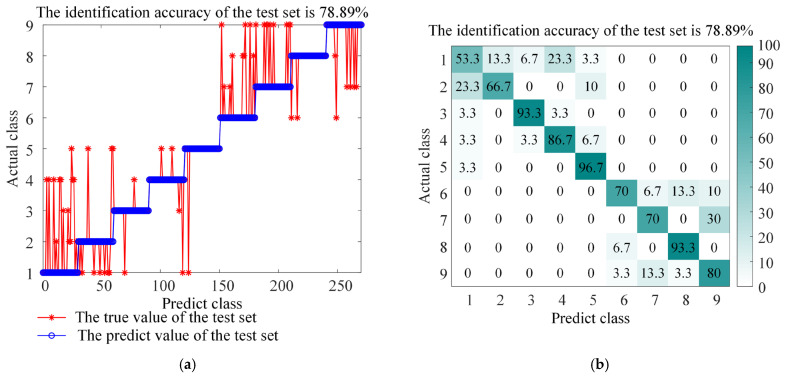

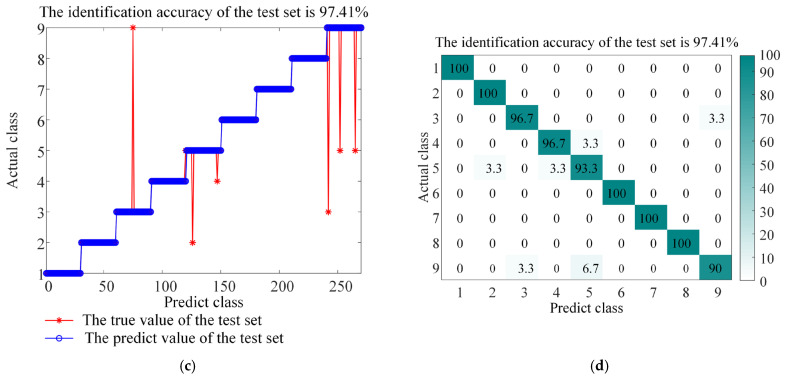
Accuracy and confusion matrix, (**a**) BAYES-ICEEMDAN-SNR-LSSVM classification accuracy, (**b**) BAYES-ICEEMDAN-SNR-LSSVM confusion matrix, (**c**) BAYES-ICEEMDAN-SNR-ITOC-LSSVM classification accuracy, and (**d**) BAYES-ICEEMDAN-SNR-ITOC-LSSVM confusion matrix.

Figure 12 shows the comparison of classification results between LSSVM and ITOC-LSSVM on the gear dataset. The experimental results show that the classification accuracy of LSSVM without optimization is 78.89% when dealing with gear faults under multiple working conditions, while the recognition rate of the model optimized by the TOC algorithm is improved to 97.04%.

The confusion matrix shown in Figure 12 further shows that ITOC-LSSVM can effectively distinguish similar impact features such as broken teeth and missing teeth, and the missed diagnosis rate and misdiagnosis rate are significantly reduced. The test results show that the BAYES-ICEEMDAN-SNR-ITOC-LSSVM model proposed in this paper is not only suitable for bearing fault diagnosis but also has high accuracy and reliability in fault identification of the gearbox under complex working conditions.

### 3.4. Ablation Experiment

In order to systematically evaluate the contribution of each core module in the BAYES-ICEEMDAN-SNR-ITOC-LSSVM framework proposed in this paper, ablation experiments are designed in this section. By constructing and testing variant models of five key components, this paper aims to quantitatively analyze the impact of adaptive noise parameter optimization (Bayesian optimization), signal decomposition method improvement, adaptive signal-to-noise ratio adjustment (SNR) and classifier parameter optimization (ITOC) on the final fault diagnosis performance.

Five ablation variants were designed in this experiment

(1) BAYES-EEMD-SNR-ITOC-LSSVM: the core decomposition method is replaced by ensemble empirical mode decomposition (EEMD) to evaluate the advantages of ICEEMDAN in suppressing mode aliasing and reducing reconstruction error.
(2) BAYES-EMD-SNR-ITOC-LSSVM: the core decomposition method is replaced by the basic empirical mode decomposition (EMD) to verify the necessity of introducing the noise-assisted integrated decomposition strategy.
(3) BAYES-ICEEMDAN-ITOC-LSSVM: remove the adaptive signal-to-noise ratio (SNR) adjustment module and adopt a fixed noise injection strategy to evaluate the impact of the dynamic SNR mechanism on the robustness of feature extraction in a strong noise environment.
(4) BAYES-ICEEMDAN-SNR-LSSVM: remove the improved Tornado Optimizer with Coriolis force (ITOC) and determine the LSSVM parameters by default or grid search to measure the value of ITOC in optimizing the classifier super parameters and avoiding local optimization.
(5) ICEEMDAN-SNR-ITOC-LSSVM: remove the Bayesian optimization module and set the noise parameters of ICEEMDAN with empirical values to verify the role of Bayesian optimization in adaptively determining the optimal noise parameters and improving the decomposition quality.

The experiment was conducted on the Case Western Reserve University (CWRU) bearing dataset, covering ten kinds of bearing health states: normal state and three kinds of fault types: inner ring fault, rolling element fault and outer ring fault. Each fault includes three kinds of defect diameters of 0.007 inch, 0.014 inch and 0.021 inch, making a total of ten types of samples. All variants use the same training/test set partition (7:3), the same permutation entropy feature extraction process (select the first eight IMF components to calculate PE) and the same evaluation index.

**Figure 13:**
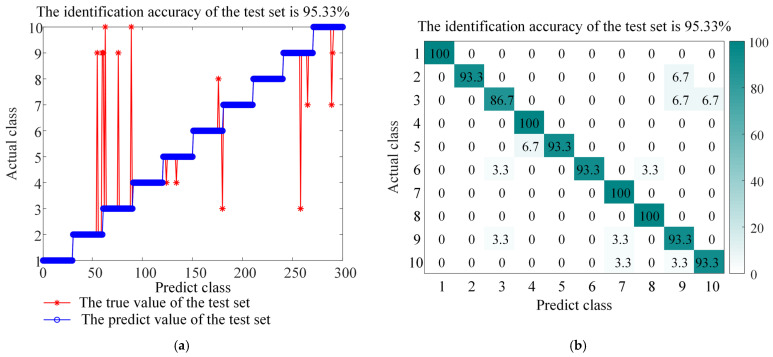
Accuracy and confusion matrix for BAYES-EEMD-SNR-ITOC-LSSVM, (**a**) BAYES-EEMD-SNR-ITOC-LSSVM classification accuracy, and (**b**) BAYES-EEMD-SNR-ITOC-LSSVM confusion matrix.

**Figure 14:**
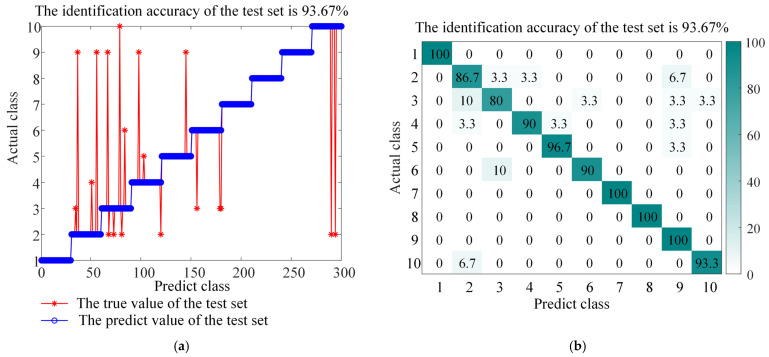
Accuracy and confusion matrix for BAYES-EMD-SNR-ITOC-LSSVM, (**a**) BAYES-EMD-SNR-ITOC-LSSVM classification accuracy, and (**b**) BAYES-EMD-SNR-ITOC-LSSVM confusion matrix.

**Figure 15:**
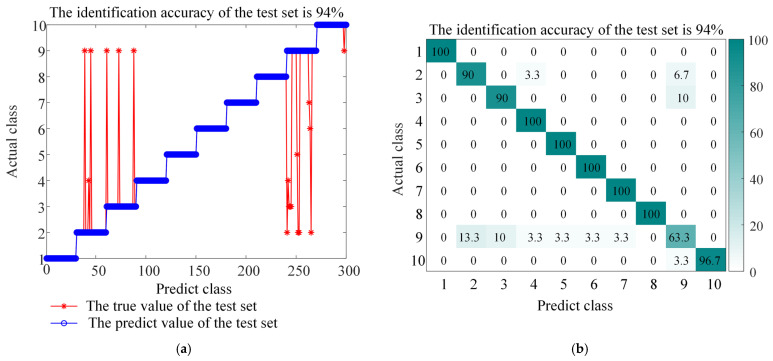
Accuracy and confusion matrix for BAYES-ICEEMDAN-ITOC-LSSVM, (**a**) BAYES-ICEEMDAN-ITOC-LSSVM classification accuracy, and (**b**) BAYES-ICEEMDAN-ITOC-LSSVM confusion matrix.

**Figure 16:**
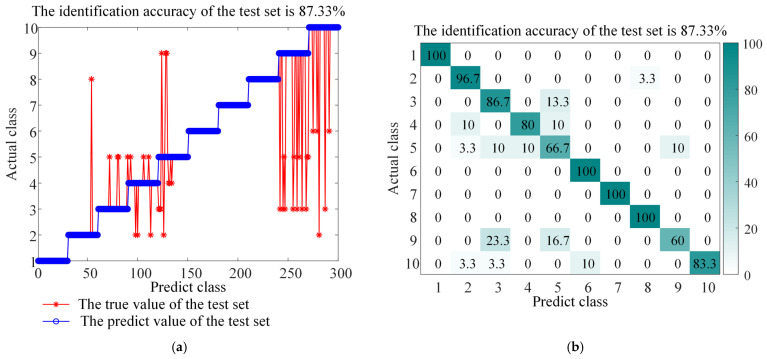
Accuracy and confusion matrix for BAYES-ICEEMDAN-SNR-LSSVM, (**a**) BAYES-ICEEMDAN-SNR-LSSVM classification accuracy, and (**b**) BAYES-ICEEMDAN-SNR-LSSVM confusion matrix.

**Figure 17:**
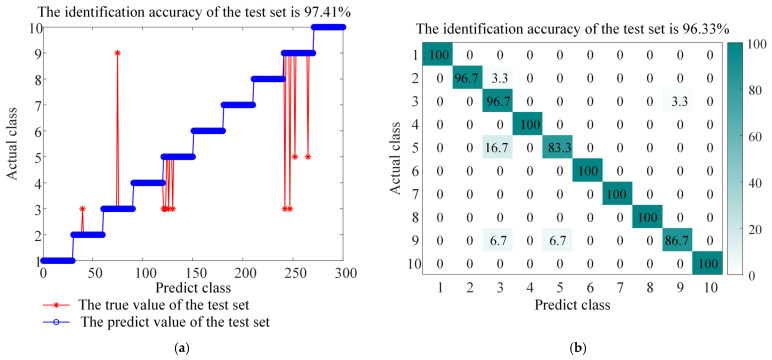
Accuracy and confusion matrix for ICEEMDAN-SNR-ITOC-LSSVM, (**a**) ICEEMDAN-SNR-ITOC-LSSVM classification accuracy, and (**b**) ICEEMDAN-SNR-ITOC-LSSVM confusion matrix.

The experimental results (as shown in Figure 13, Figure 14, Figure 15, Figure 16 and Figure 17 and the corresponding confusion matrix) clearly show that the complete BAYES-ICEEMDAN-SNR-ITOC-LSSVM model achieves the highest fault diagnosis accuracy (97.67%) compared with any ablation variant. Specific analysis is as follows:

Variants (1) and (2): When EEMD or EMD is used to replace ICEEMDAN, the accuracy is significantly reduced. This confirms that ICEEMDAN has advantages in suppressing mode aliasing and residual noise when dealing with the non-stationary and nonlinear characteristics of bearing vibration signals, and provides a higher quality intrinsic mode function (IMF) component for subsequent feature extraction.

Variant (3): After removing the adaptive SNR adjustment, the model is unstable in the signal segment where the noise intensity changes, and the accuracy is reduced. This highlights the key role of the dynamic SNR mechanism, which can adjust the noise injection intensity in real time according to the local signal-to-noise ratio of the signal, so as to enhance the weak fault characteristics more effectively under the strong background noise.

Variant (4): When the LSSVM parameters are optimized without ITOC, the classification accuracy decreases most significantly. This proves that the ITOC algorithm effectively improves the global search ability and convergence accuracy by integrating the Chebyshev chaotic map, Cauchy mutation and a dynamic reverse learning strategy. The LSSVM super parameter optimized by the ITOC algorithm significantly improves the classification boundary and generalization performance of the model.

Variant (5): After using fixed noise parameters, the performance of the model also declines. This shows that the Bayesian optimization module can efficiently and automatically search for the optimal noise parameters of ICEEMDAN, avoid the uncertainty of manual trial and error, and ensure the adaptability and stability of the signal decomposition process in different operating states.

The experimental results show that each improved module in the proposed framework is indispensable. BAYES-ICEEMDAN-SNR module works together to realize adaptive, highly robust decomposition and feature enhancement of vibration signal in a strong noise environment; the ITOC module significantly improves the parameter optimization efficiency and final performance of LSSVM classifier. All modules work together to form the basis of a high-precision and strong adaptive fault diagnosis method in this paper.

### 3.5. Discussion

The experimental results show that the proposed BAYES-ICEEMDAN-SNR-ITOC-LSSVM method achieves a competitive fault diagnosis accuracy of 97.67% on the CWRU dataset. We note that some studies in the literature have reported that the accuracy rate is close to 100% on the benchmark dataset. This raises a reasonable question: why do we still need such a complex method as we proposed?

The answer lies in the core objectives and practical considerations of our research, which go beyond the pursuit of the highest accuracy only on specific and well-structured public datasets. Our method design focuses on robustness, adaptability and engineering practicability in challenging environments, especially in scenes with strong noise and variable noise, which can better reflect the real industrial environment. Its contribution and design reasons are detailed as follows:

Emphasize robustness in a strong noise environment: Many methods that achieve high accuracy on CWRU may use carefully preprocessed data, optimal signal segmentation or highly optimized features for specific noise characteristics of the dataset. In contrast, the core innovation of our BAYES-ICEEMDAN-SNR module lies in its ability to achieve adaptive denoising and feature enhancement under strong noise. Integrating Bayesian optimization and a dynamic SNR adjustment mechanism, it is specially used to deal with signal quality changes without human intervention. The 97.67% accuracy rate reported in this paper was obtained under a consistent and possibly more challenging evaluation framework (for example, using sliding windows and randomly dividing datasets under various operating conditions), showing its reliable performance rather than the peak performance under ideal settings.

Advantages within the comparable framework: As shown in Table 5, under the same experimental settings, our method (97.67%) is superior to other representative diagnostic models in the same period (for example, 95.53% of WEST-ICNN-IHBA-LSSVM and 94.04% of EMD-INGO-LSSVM). This comparative analysis verifies that the proposed integration framework, which combines the adaptive signal processor with the improved optimizer, is more effective than other complex hybrid methods in the same comparison range.

Provide end-to-end adaptive solutions: A major challenge in industrial applications is the lack of universally optimal parameter sets. The method to achieve 100% accuracy usually depends on a large number of manual parameter tuning for the CWRU dataset. Our work contributes to an end-to-end process embedded with adaptive mechanisms (BAYES-ICEEMDAN-SNR for signal processing and ITOC for classifier tuning). This reduces the dependence on expert knowledge and the trial and error method and enhances the generalization ability of the method to different machines or working conditions, because the noise level and signal characteristics of these scenes may be different from the CWRU benchmark.

Lay the foundation for handling more complex scenarios: the CWRU dataset is the benchmark for verifying the functions of the core algorithm. The architecture of the proposed method is more suitable for dealing with scenes involving non-stationary noise, variable speed or limited marker data, which are common challenges in predictive maintenance in the real world. The high accuracy obtained on CWRU confirms the solid foundation of this method, and its adaptive design enables it to deal with more complex and less manual industrial data.

In summary, although the peak accuracy is an important indicator, the engineering significance of the diagnosis method also includes its adaptability, robustness and deployment convenience. The proposed BAYES-ICEEMDAN-SNR-ITOC-LSSVM framework provides a balanced and precise solution. It not only provides high accuracy but also solves the key practical challenges in automatic fault diagnosis, which proves the rationality of its design complexity.

## 4. Conclusions

In view of the difficulties in extracting vibration signal features in the fault diagnosis of rolling bearings, the low efficiency of optimizing diagnostic model parameters, and the tendency to fall into local optima, a fault diagnosis method for rolling bearings based on wavelet transformation is proposed. A fault diagnosis method based on BAYES-ICEEMDAN-SNR feature extraction and ITOC-LSSVM is proposed. Through theoretical analysis of the system, algorithm improvement and experimental verification, the following conclusions are drawn:

(1) By introducing Bayesian optimization and an adaptive signal-to-noise ratio adjustment mechanism, the ICEEMDAN algorithm has been significantly improved, effectively overcoming the dependence of the original algorithm on the empirical setting of noise parameters. Bayesian optimization enables adaptive global optimization of the noise standard deviation, significantly enhancing the stability and adaptability of the decomposition process. This algorithm combines real-time dynamic assessment based on signal-to-noise ratio and noise intensity adjustment, enabling adaptive adjustment of the decomposition strategy according to different signal qualities, enhancing the ability to extract fault features while suppressing over-decomposition and mode aliasing. The improved BAYES-ICEEMDAN-SNR further improves the decomposition quality and feature representation robustness of non-stationary and nonlinear vibration signals.
(2) The improved tornado optimization algorithm exhibits excellent global optimization performance. By incorporating Chebyshev chaotic mapping initialization, Cauchy mutation strategy, and a dynamic reverse learning mechanism, ITOC effectively enhances population diversity, global search capability, and the ability to escape from local optima, overcoming the shortcomings of the original TOC algorithm, which is prone to premature convergence and easily trapped in local optima. Simulation results on 12 standard test functions indicate that ITOC outperforms the original TOC algorithm and comparison algorithms such as GWO, WOA, and NGO in terms of convergence speed, solution accuracy, and stability.
(3) The LSSVM diagnostic model jointly optimized by BAYES-ICEEMDAN-SNR and ITOC has high recognition accuracy and engineering practicability. The permutation entropy features extracted by the improved BAYES-ICEEMDAN-SNR algorithm can more comprehensively characterize different fault states; the ITOC algorithm adaptively optimizes the key parameters of LSSVM to construct a high-performance fault classifier. The verification on the bearing dataset of Case Western Reserve University and the gear dataset of Huazhong University of Science and Technology shows that the overall fault diagnosis accuracy of this method reaches over 97%, significantly higher than that of the mainstream diagnostic models such as standard LSSVM. These experimental results prove the effectiveness of this method in extracting fault features and achieving accurate classification in noisy environments, and it has a good engineering application prospect.

Although the proposed BAYES-ICEEMDAN-SNR-ITOC-LSSVM framework demonstrated superior diagnostic accuracy and robustness on the CWRU dataset, it still has certain limitations that require further investigation:

(1) Higher computational burden: The integration of Bayesian optimization with the ITOC algorithm increases the complexity of the hyperparameter tuning stage. Although this ensures higher accuracy, the computational cost of the offline training phase is higher than that of non-optimized models.
(2) Generalization ability under dynamic conditions: The current verification is mainly based on stable operation conditions. The performance and reliability of this framework in strong non-stationary environments (such as rapid load fluctuations or time-varying rotational speeds) still need to be comprehensively evaluated.
(3) Empirical feature selection: The selection of the first eight IMFs for the calculation of permutation entropy relies on prior knowledge of the bearing fault characteristics. Developing an automated, data-driven criterion for the optimal selection of IMF will enhance the adaptability and universality of the diagnostic process.

Future research will focus on enhancing the computational efficiency of the ITOC algorithm to support real-time monitoring and will expand this framework to the field of cross-domain transfer learning to address the challenge of scarce data in practical industrial applications.

## Figures and Tables

**Table 1:** Test Function.

Function	Function Name	Search Range	Theoretical Optimum
F1	Sphere	[−100, 100] ^n^	0
F2	Schwefel 2.22	[−10, 10] ^n^	0
F3	Schwefel 1.2	[−100, 100] ^n^	0
F4	Schwefel 2.21	[−100, 100] ^n^	0
F5	Quartic	[−1.28, 1.28] ^n^	0
F6	Schwefel	[−500, 500] ^n^	−12,569.5
F7	Rastrigin	[−5.12, 5.12] ^n^	0
F8	Ackley	[−32, 32] ^n^	0
F9	Griewank	[−600, 600] ^n^	0
F10	Shekel’s Foxholes	[−65.536, 65.536] ^n^	1
F11	Three-Hump Camel	[−5, 5] ^n^	−1.0316
F12	Branin	[−5, 10] × [0, 15]	0.398

**Note:** n represents the number of independent variables.

**Table 2:** Test Function Run Results.

Function	Algorithm	Avg	Std	Function	Algorithm	Avg	Std	Function	Algorithm	Avg	Std
F1	ITOC	0	0	F2	ITOC	0	0	F3	ITOC	0	0
	TOC	6.4 × 10^−11^	8.05 × 10^−11^		TOC	0.0019	0.0023		TOC	5.05 × 10^−9^	7.14 × 10^−9^
	GWO	3.1 × 10^−23^	1.13 × 10^−34^		GWO	3.79 × 10^−13^	2.67 × 10^−19^		GWO	2.46 × 10^−13^	0.00017
	WOA	1.07 × 10^−34^	3.74 × 10^−27^		WOA	2.65 × 10^−19^	1.15 × 10^−13^		WOA	0.000120	5.01 × 10^−18^
	NGO	6.02 × 10^−27^	3.74 × 10^−27^		NGO	3.46 × 10^−13^	1.15 × 10^−13^		NGO	1.39 × 10^−17^	5.01 × 10^−18^
F4	ITOC	0	0	F5	ITOC	0.002090	0.001964	F6	ITOC	−1.3 × 10^61^	1.84 × 10^61^
	TOC	0.02259	0.03182		TOC	0.175270	0.068869		TOC	−6695.97	178.335
	GWO	1.38 × 10^−9^	0.12584		GWO	0.012965	2.41 × 10^−3^		GWO	−6074.69	901.897
	WOA	0.08907	1.61 × 10^−13^		WOA	0.007828	0.009914		WOA	−10, 474.2	2849.55
	NGO	8.55 × 10^−13^	1.61 × 10^−13^		NGO	0.003960	0.002343		NGO	−4668.43	174.470
F7	ITOC	0	0	F8	ITOC	0	0	F9	ITOC	0	0
	TOC	179.6657	32.59126		TOC	0.0105	0.0149		TOC	24.4656	4.93554
	GWO	15.46264	2.191846		GWO	0.2078	0.0093		GWO	0.036864	0.00159
	WOA	128.1135	181.1798		WOA	3.33 × 10^−16^	1.57 × 10^−16^		WOA	1.67 × 10^−16^	2.36 × 10^−16^
	NGO	4.55 × 10^−13^	4.82 × 10^−13^		NGO	327.9205	60.9416		NGO	1.24 × 10^−13^	6.85 × 10^−14^
F10	ITOC	1.001315	0.004389	F11	ITOC	−1.03093	0.000405	F12	ITOC	0.400634	0.00382
	TOC	1.495017	0.702883		TOC	−1.03163	7.57 × 10^−14^		TOC	0.397887	0
	GWO	2.487068	0.700088		GWO	−1.03163	9.82 × 10^−7^		GWO	0.397902	1.81 × 10^−5^
	WOA	5.880597	6.905014		WOA	−1.03163	7.73 × 10^−7^		WOA	0.397890	2.1 × 10^−6^
	NGO	0.998003	9.33 × 10^−8^		NGO	−1.03163	7.8 × 10^−13^		NGO	0.397887	1.45 × 10^−11^

**Table 3:** The main parameters of the test bearing.

Pitch Diameter D/mm	Ball Diameter d/mm	Number of Balls z	Contact Angle β/(°)
39.04	7.94	9	0

**Table 4:** Setting parameters for the BAYES-ICEEMDAN-SNR algorithm.

Parameter	Set Value
*Nstd*	0.1
NR	80
MaxIter	8
SNRFlag	1
M	1
T	3

**Table 5:** Classification accuracy of different models.

Classification Model	Accuracy
TRP-SVD-SVM	92.71%
RP-SVD-SVM	73.00%
WEST-ICNN-IHBA-LSSVM	95.53%
EMD-INGO-LSSVM	94.04%
LSSVM	84%
ITOC-LSSVM	97.67%

**Table 6:** Operating Condition.

Operating Condition	Rotating Speed/Hz	Load/Nm
1	20	0.113
2	25	0.226
3	30	0.339
4	35	0.452

## Data Availability

The raw data supporting the conclusions of this article will be made available by the authors on request.

## References

[1] Li Y., Liu X., Hu J., Liang P., Wang B., Yuan X., Zhang L. (2025). Graph Optimization Algorithm Enhanced by Dual-Scale Spectral Features with Contrastive Learning for Robust Bearing Fault Diagnosis. Knowl. Based Syst..

[2] Zhao H., Huang X., Xiao Z., Shi H., Li C., Tai Y. (2024). Week-Ahead Hourly Solar Irradiation Forecasting Method Based on ICEEMDAN and TimesNet Networks. Renew. Energy.

[3] Xiao M., Wang Z., Zhao Y., Geng G., Dustdar S., Donta P.K., Ji G. (2023). A New Fault Feature Extraction Method of Rolling Bearings Based on the Improved Self-Selection ICEEMDAN-Permutation Entropy. ISA Trans..

[4] Yuvaraju B.A.G., Srinivas J., Ioannou I., Guruguntla V., Ghantasala G.S.P. (2025). Advanced Chatter Detection in Internal Turning for Industry 4.0: Adaptive Threshold Wavelet De-Noising with Enhanced ICEEMDAN–Hilbert Fusion Using Adaptive Probabilistic Neural Network. J. Manuf. Process..

[5] Xiang G., Sun A., Liu Y., Gao L. (2024). An Improved Denoising Method for φ-OTDR Signal Based on the Combination of Temporal Local GMCC and ICEEMDAN-WT. Opt. Fiber Technol..

[6] Zhang T., Gu Y., Zhang Q., Wei Y., Wang L., Li C., Wang W., Lv J., Feng Y. (2025). Performance Improvement of Quartz-Enhanced Photoacoustic Spectroscopy Gas System Using ICEEMDAN-PE-WTD. Infrared Phys. Technol..

[7] Zhong J., Cai Y., Wang K., Chen Y., Wei X., Su H., Zheng X., Liang K. (2025). Two-Stage Hybrid Energy Storage Configuration Method for Distribution Networks Based on ICEEMDAN and Multi-Objective Optimization. J. Energy Storage.

[8] Guo H., Zhao X. (2026). An Interpretable Fault Diagnosis Method for Marine Rotating Machinery under Strong Noise Based on Constrained Penalty Leech Algorithm Optimized ICEEMDAN. Ocean Eng..

[9] Song Z., Guo X., Zhang W., Deng Y., Li Z., Fan G., Kang R., Wang X. (2025). Atomic-Scale Surface Integrity Prediction of Chemical Mechanical Polishing Polycrystalline Diamond: Insights from ReaxFF MD and CPO-GMM-LSSVM Model. Diam. Relat. Mater..

[10] Yang J., Guan H., Ma X., Zhang Y., Lu Y. (2025). Rapid Detection of Corn Moisture Content Based on Improved ICEEMDAN Algorithm Combined with TCN-BiGRU Model. Food Chem..

[11] Yang J., Peng L., Luo L., Li W., Chen Y. (2025). Wind Power Forecasting Using Hybrid ICEEMDAN-KPCA and IWOA-BiLSTM Models. Int. J. Electr. Power Energy Syst..

[12] Zhou M., Yu J., Wang M., Quan W., Bian C. (2024). Research on the Combined Forecasting Model of Cooling Load Based on IVMD-WOA-LSSVM. Energy Build..

[13] Zhu Z., Liu H., Lei W., Xue Y., Xiao C. (2025). Optimising Wellbore Annular Leakage Detection and Diagnosis Model: A Signal Feature Enhancement and Hybrid Intelligent Optimised LSSVM Approach. Mech. Syst. Signal Process..

[14] Zhao Y., Jiang A., Jiang W., Zhang Y., Zhang S. (2025). Hydraulic Pump Fault Diagnosis by Modified Slime Mold Algorithm Optimized Support Vector Machine. Measurement.

[15] Xia Y., Zhao H., Feng X. (2025). Prediction of Grease Performance and Optimal Additive Ratio Based on the SSA-GDA-LSSVM Model. Tribol. Int..

[16] Wang W., Wang H., Li X., Qi Y., Cui X., Bai C. (2026). Prediction Model of Coal Spontaneous Combustion Oxidation State Based on MICPO-LSSVM. Fuel.

[17] Liu J., Song Y., Yu X. (2024). Risk Assessment Study of Hydrogen Energy Storage System Based on KPCA-TSO-LSSVM. Int. J. Hydrogen Energy.

[18] Wang H., Chen X., Xia J., Zhao H., Maddaiah P.N. (2026). Newton-Raphson-Based Optimizer Combined with LSSVM: Temperature Compensation Applied to Small-Range Electronic Pressure Scanners. Flow Meas. Instrum..

[19] Han A., Uranghai X., Mei L., Dong Y., Yan G., Huang A., Bao Y., Qing S., Jukov A., Mandakh U. (2026). Inversion of low heavy metal content in Soil-Scutellaria baicalensis systems using optimized spectral indices and LSSVM. Int. J. Appl. Earth Obs. Geoinf..

[20] Meng S., Shi Z., Gutierrez M. (2025). Interpretable CEEMDAN-SMA-LSSVM Hybrid Model for Predicting Shield Tunnel-Induced Settlement. J. Rock Mech. Geotech. Eng..

[21] Wang W., Zhang X., Tian W., Li Z., Zhang R. (2025). Development and Evaluation of a Novel DTIB-LSSVM Model for Efficient and Accurate Runoff Forecasting. Results Eng..

[22] Huang K., Li W., Gao F. (2025). Barabási-Albert Model-Enhanced Genetic Algorithm for Optimizing LGBM in Ship Power Grid Fault Diagnosis. Measurement.

[23] Zhu Q., Liu X., Pei S., Chai S., Chai R., Cui L. (2025). An Optimization Method of Low Orbit Constellation Satellite Fault Diagnosis Based on Improved Artificial Bee Colony Algorithm. IFAC-Pap..

[24] Wang S., Yi S., Zhao B., Li Y., Zhang D., Chen T., Sun W. (2025). Metaheuristic-Optimized LSSVM Integrated with Multi-Modal Sensing for Intelligent Nutrient Monitoring in Saline-Alkali Soils. J. Environ. Chem. Eng..

[25] Farokhzadeh B., Choobeh S., Imani R. (2026). Robust Monthly Precipitation Forecasts Using VMD-Decomposed Hybrid LSSVM–BiLSTM Model Optimized by Harris Hawks Algorithm. Phys. Chem. Earth Parts A/B/C.

[26] Quan R., Liang W., Wang J., Li X., Chang Y. (2024). An Enhanced Fault Diagnosis Method for Fuel Cell System Using a Kernel Extreme Learning Machine Optimized with Improved Sparrow Search Algorithm. Int. J. Hydrogen Energy.

[27] Dong Z., Ding Y., Liu W., Hu Z., Lu S., Liu Y. (2025). Maneuverability Parameter Identification of a Water-Jet USV Based on Truncated Weighted LSSVM Integrated with Adaptive Mutation PSO Algorithm. Ocean Eng..

[28] Huang K., Li W., Fang H., Wu X., Wang L., Peng H. (2024). IPORF: A Combined Improved Parrot Optimizer Algorithm and Random Forest for Fault Diagnosis in AUV. Ocean Eng..

[29] Hajji M., Hichri A., Yahyaoui Z., Mansouri M., Bouazzi Y., Rajhi W., Ahmad A. (2024). Reducing Neural Network Complexity via Optimization Algorithms for Fault Diagnosis in Renewable Energy Systems. Ain Shams Eng. J..

[30] Dao F., Zeng Y., Qian J. (2024). Fault Diagnosis of Hydro-Turbine via the Incorporation of Bayesian Algorithm Optimized CNN-LSTM Neural Network. Energy.

[31] Chen Y., Wang J., Chen W., Dao F., Zeng Y., Lv S. (2024). Fault Diagnosis in Hydropower Units Based on Chaotic Kepler Optimization Algorithm-Enhanced BiLSTM Model. Energy Rep..

[32] Ji C., Zhang C., Suo L., Liu Q., Peng T. (2024). Swarm Intelligence Based Deep Learning Model via Improved Whale Optimization Algorithm and Bi-Directional Long Short-Term Memory for Fault Diagnosis of Chemical Processes. ISA Trans..

[33] Torres M.E., Colominas M.A., Schlotthauer G., Flandrin P. A complete ensemble empirical mode decomposition with adaptive noise. Proceedings of the 2011 IEEE International Conference on Acoustics, Speech and Signal Processing (ICASSP).

[34] Braik M., Al-Hiary H., Alzoubi H., Hammouri A., Azmi Al-Betar M., Awadallah M.A. (2025). Tornado Optimizer with Coriolis force with Coriolis Force: A Novel Bio-Inspired Meta-Heuristic Algorithm for Solving Engineering Problems. Artif. Intell. Rev..

[35] Kocarev L., Jakimovski G. (2001). Chaos and cryptography: From chaotic maps to encryption algorithms. IEEE Trans. Circuits Syst. I.

[36] Yao X., Liu Y. (1996). Fast Evolutionary Programming. Evol. Program..

[37] Rahnamayan S., Tizhoosh H.R., Salama M.M. (2008). Opposition-based differential evolution. IEEE Trans. Evol. Comput..

[38] Smith W.A., Randall R.B. (2015). Rolling element bearing diagnostics using the Case Western Reserve University data: Abenchmark study. Mech. Syst. Signal Process..

[39] Zhao C., Zio E., Shen W. (2024). Domain generalization for cross-domain fault diagnosis: An application-oriented perspective and a benchmark study. Reliab. Eng. Syst. Saf..

